# Formation of Polyglutamine Inclusions in a Wide Range of Non-CNS Tissues in the *Hdh*Q150 Knock-In Mouse Model of Huntington's Disease

**DOI:** 10.1371/journal.pone.0008025

**Published:** 2009-11-30

**Authors:** Hilary Moffitt, Graham D. McPhail, Ben Woodman, Carl Hobbs, Gillian P. Bates

**Affiliations:** 1 Department of Medical and Molecular Genetics, King's College London School of Medicine, London, United Kingdom; 2 Division of Cellular Pathology, Barts and the London NHS Trust, Royal London Hospital, London, United Kingdom; 3 Wolfson Centre for Age-Related Diseases, Kings College London School of Medicine, London, United Kingdom; Tokyo Medical and Dental University, Japan

## Abstract

**Background:**

Huntington's disease (HD) is an inherited progressive neurodegenerative disorder caused by a CAG repeat expansion in the ubiquitously expressed *HD* gene resulting in an abnormally long polyglutamine repeat in the huntingtin protein. Polyglutamine inclusions are a hallmark of the neuropathology of HD. We have previously shown that inclusion pathology is also present in the peripheral tissues of the R6/2 mouse model of HD which expresses a small N-terminal fragment of mutant huntingtin. To determine whether this peripheral pathology is a consequence of the aberrant expression of this N-terminal fragment, we extend this analysis to the genetically precise knock-in mouse model of HD, *Hdh*Q150, which expresses mutant mouse huntingtin.

**Methodology/Principal Findings:**

We have previously standardized the CAG repeat size and strain background of the R6/2 and *Hdh*Q150 knock-in mouse models and found that they develop a comparable and widespread neuropathology. To determine whether *Hdh*Q150 knock-in mice also develop peripheral inclusion pathology, homozygous *Hdh* ^Q150/Q150^ mice were perfusion fixed at 22 months of age, and tissues were processed for histology and immunohistochemistry with the anti-huntingtin antibody S830. The peripheral inclusion pathology was almost identical to that found in R6/2 mice at 12 weeks of age with minor differences in inclusion abundance.

**Conclusions/Significance:**

The highly comparable peripheral inclusion pathology that is present in both the R6/2 and *Hdh*Q150 knock-in models of HD indicates that the presence of peripheral inclusions in R6/2 mice is not a consequence of the aberrant expression of an N-terminal huntingtin protein. It remains to be determined whether peripheral inclusions are a pathological feature of the human disease. Both mouse models carry CAG repeats that cause childhood disease in humans, and therefore, inclusion pathology may be a feature of the childhood rather than the adult forms of HD. It is important to establish the extent to which peripheral pathology causes the peripheral symptoms of HD from the perspective of a mechanistic understanding and future treatment options.

## Introduction

Huntington's disease (HD) is an autosomal dominant late-onset progressive neurodegenerative disorder with a mean age of onset of 40 years. Symptoms include motor disorders, psychiatric disturbances, cognitive decline and weight loss. Disease duration is 15 –20 years and there are no effective disease modifying treatments [1]. The HD mutation is an expanded CAG repeat in the *HD* gene that is translated into a polyglutamine (polyQ) repeat in the huntingtin (Htt) protein [2]. Unaffected individuals have (CAG)_6–35_ repeats, whilst disease causing alleles of (CAG)_40_ and above are fully penetrant [3], [4]. Age of symptom onset can range from early childhood to extreme old age with repeats of (CAG)_75_ and above invariably causing the childhood form of the disease [4]. Neuropathologically, the disease is characterized by global brain atrophy [5], [6], neuronal cell loss in the striatum, cortex and other brain regions and the deposition of nuclear and cytoplasmic polyQ aggregates [7], [8].

Mouse models of HD include transgenic mice that express either N-terminal fragments of, or full-length human Htt, as well as the genetically precise knock-in models, in which CAG repeats in the mutant range have been inserted into the mouse HD gene (*Hdh*) [9]. The N-terminal fragment models [10], [11], develop early onset HD-related phenotypes with rapid progression which, especially in the case of the R6/2 mouse, has allowed extensive complementary analyses and established this model as a screening tool. We recently conducted a comparison of R6/2 (age 12 weeks) and homozygous *Hdh*Q150 knock-in (*Hdh* ^Q150/Q150^ aged 22 months) mice [12], [13] at late stage disease (with relatively standardised strain background and CAG repeat size) and found that both models exhibited widespread and comparable brain phenotypes. Nuclear inclusions and neuropil aggregates were distributed throughout all brain regions in both models [13]. Microarray expression profiles from both striatum [14] and cerebellum (Luthi-Carter and Bates, unpublished data) of the R6/2 and *Hdh* ^Q150/Q150^ mice were highly correlated. We had attributed these widespread pathologies in the R6/2 mouse brain to the expression of a small N-terminal fragment of Htt and therefore were surprised to find that it was also a feature of the *Hdh* ^Q150/Q150^ mice.

We have previously reported that polyQ aggregate pathology, in the form of nuclear inclusions, is also present in a wide range of peripheral tissues in the R6/2 mouse [15] including skeletal muscle and pancreatic islets. Very little is known about whether HD-related pathologies develop in the peripheral tissues of other mouse models of HD, although it has recently been reported that nuclear inclusions are present in the pancreatic islets of the N171-82Q N-terminal fragment model [16]. The peripheral pathogenesis of HD is of interest because a number of HD symptoms, that could be caused by a peripheral pathology, have been reported including weight loss [17], [18], abnormal skeletal muscle energy metabolism [19], muscle weakness [20] insulin sensitivity [21] and mitochondrial abnormalities in lymphocytes [22], [23]. In general, peripheral organs from HD patients have not been collected at *post mortem* and, unless amenable to biopsy, are not available for analysis. To begin to determine whether the peripheral aggregate pathology present in the R6/2 mice has relevance for the human disease, we have conducted a comprehensive investigation of the distribution of polyQ aggregates in the peripheral tissues of the *Hdh*Q150 knock-in model expressing mutant full length Htt at endogenous levels. We find an identical distribution in aggregate pathology between the R6/2 and *Hdh*Q150 models, with only minor differences in aggregate abundance.

## Results

**Figure 1:**
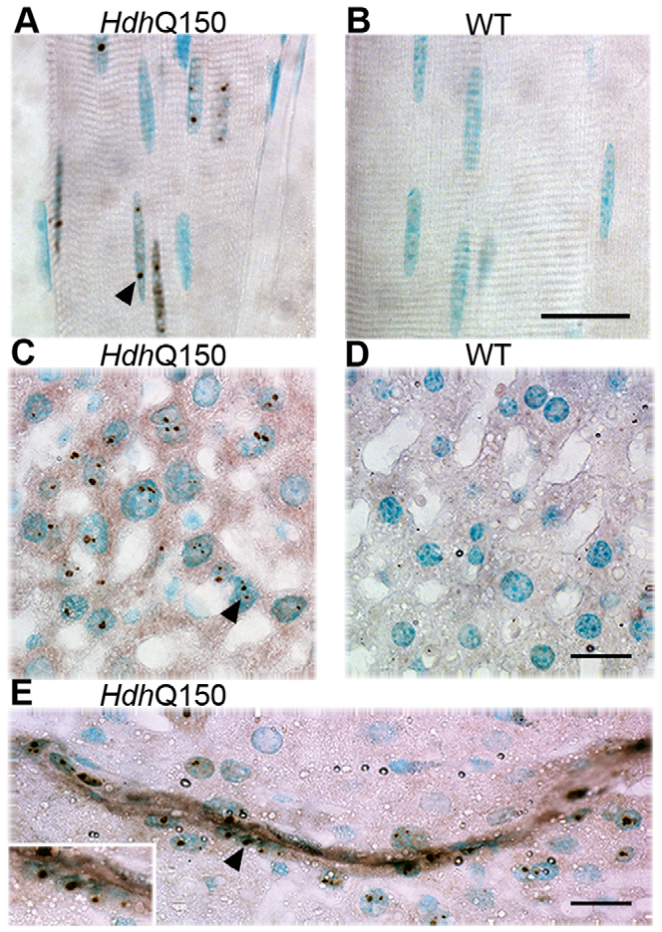
Nuclear inclusions in the skeletal muscle and liver of *Hdh* ^Q150/Q150^ mice. Nuclear inclusions in longitudinal sections of quadriceps muscle are present in *Hdh* ^Q150/Q150^ (A) but absent from wild-type (B) mice. Nuclear inclusions are present in hepatocytes from *Hdh* ^Q150/Q150^ (C) but not in those from wild-type (D) mice and are abundant in an *Hdh* ^Q150/Q150^ intrahepatic bile duct (E). The insert shows a higher magnification of the epithelial cells of the bile duct. Nuclear inclusions are indicated by arrowheads. Scale bar = 20 µm.

**Figure 2:**
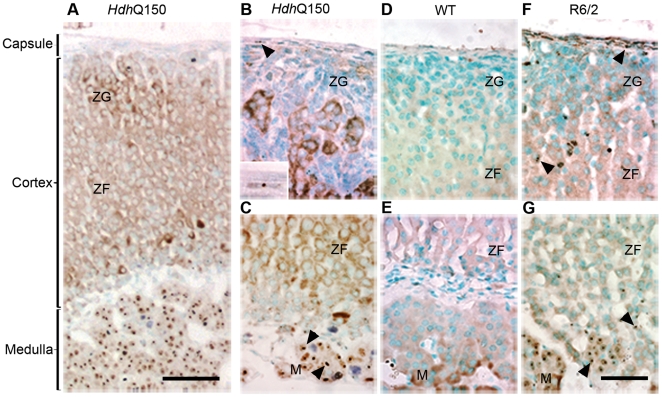
Distribution of nuclear inclusions in the adrenal glands of the *Hdh* ^Q150/Q150^ and R6/2 mice. (A) transverse section from an *Hdh* ^Q150/Q150^ mouse illustrating the structure of the adrenal gland. Inclusions present in the outer cortex (B) and inner cortex and medulla (C) from *Hdh* ^Q150/Q150^ mice. Insert in (B) shows an inclusion in a fibroblast from the adrenal capsule. Inclusions are absent from the *Hdh* ^Q150/Q150^ wild type control (D,E). A comparable distribution of inclusions is present in the R6/2 mouse (F,G) except for a greater density of inclusions in the cortex. Nuclear inclusions are indicated by arrowheads. ZG = zona glomerulosa, ZF = zona fasciculata, M = medulla. Scale bar (A) = 100 µm, scale bar (B–G) = 50 µm.

**Figure 3:**
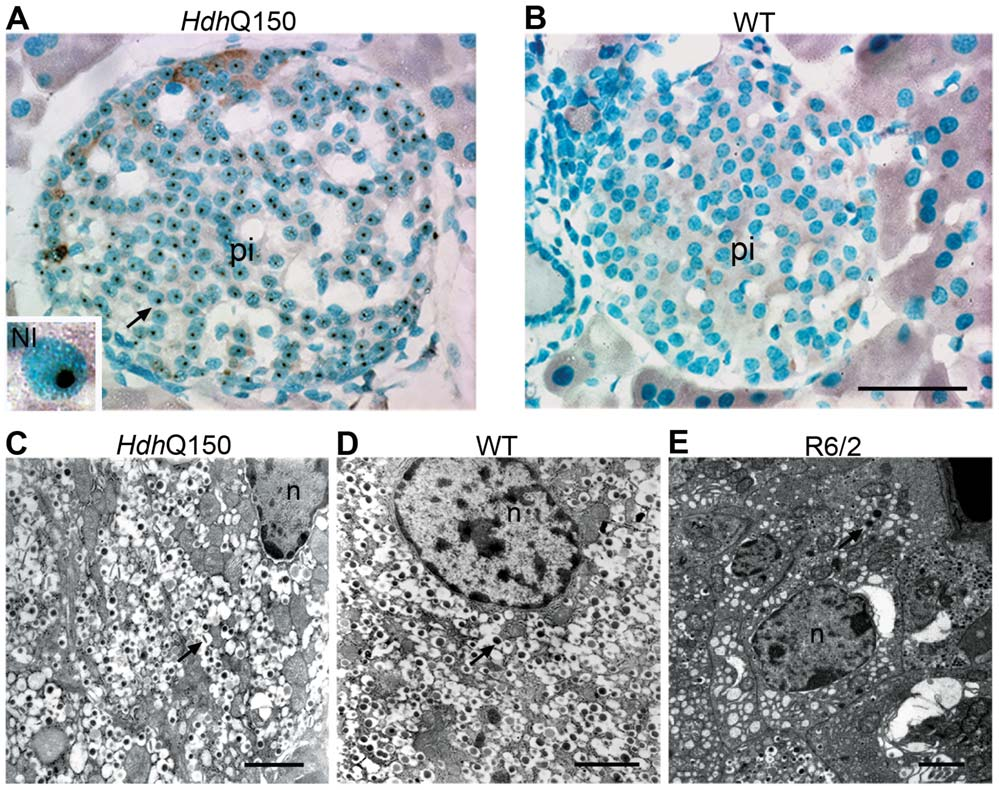
Pancreatic pathology in the *Hdh*Q150 mouse model. Nuclear inclusions were present in the pancreatic islets of *Hdh* ^Q150/Q150^ mice (A) and absent from wild-type controls (B). Insert shows higher magnification image of a nucleus containing a single inclusion indicated by arrow. Electron micrograph showing cytoplasmic detail of β-cells in the islets from an *Hdh* ^Q150/Q150^ mouse (C), a wild-type control (D), and an R6/2 mouse (E). The *Hdh* ^Q150/Q150^ β-cell appears normal as compared to the control. The R6/2 β-cell is atrophied and degranulated in comparison. Arrows indicate β-granules (C–E). Pi = pancreatic islet, NI = nuclear inclusion. Scale bar (A,B) = 50 µm, scale bar (C,D) = 1 µm, scale bar (E) = 2 µm.

**Figure 4:**
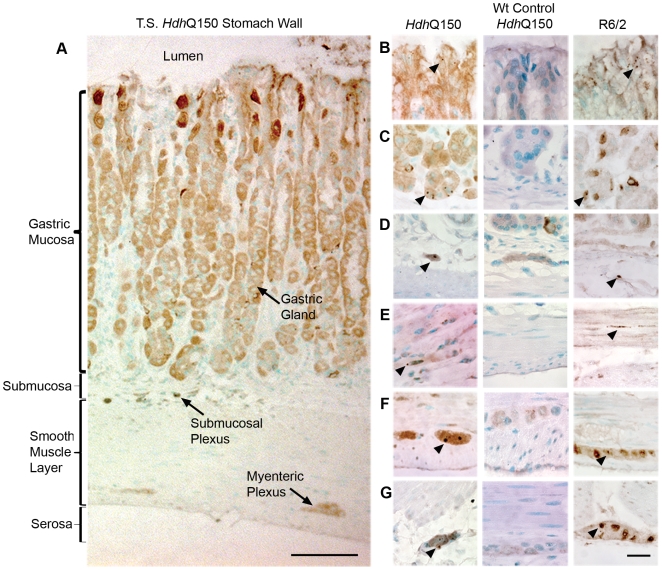
Inclusion distribution across the stomach wall in the *Hdh* ^Q150/Q150^ mice. (A) Transverse section through the stomach wall of an *Hdh* ^Q150/Q150^ mouse showing structural detail. Inclusions were present in the mucus cells in the gastric gland neck (B), the basal cells of the gastric gland (C), the submucosal ganglia (D), smooth muscle (E), the myenteric ganglia (F), and the serosal ganglia (G) of *Hdh* ^Q150/Q150^ but absent from the wild-type control. A similar distribution was found in R6/2 in comparison to the *Hdh* ^Q150/Q150^. Nuclear inclusions are indicated by arrowheads. Scale bar (A) = 100 µm, scale bar (B–G) = 20 µm.

**Figure 5:**
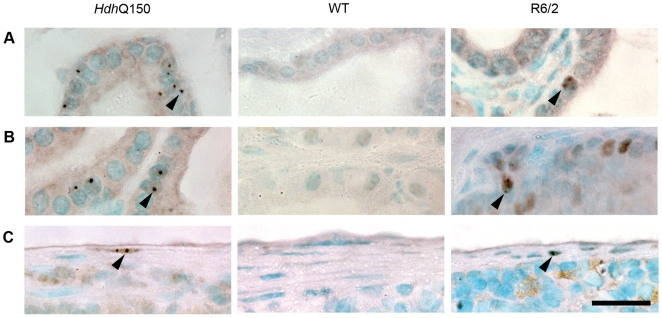
Inclusions are present in the male reproductive glandular epithelium and spleen connective tissue. Nuclear inclusions were present in the epithelial cells of the seminal vesicles (A) and the epithelium of the coagulation gland (B) in *Hdh* ^Q150/Q150^ but absent from wild-type mice. A similar distribution was identified in R6/2 mice although the number of affected cells was fewer in number in both cases. Nuclear inclusions were present in connective tissue cells of the spleen capsule from *Hdh* ^Q150/Q150^ mice (C) with a similar distribution in R6/2. Nuclear inclusions are indicated by arrowheads. Scale bar = 20 µm.

In order to investigate the distribution of inclusions in non-CNS tissue, 22 month old *Hdh* ^Q150/Q150^ mice and littermate controls were perfusion fixed and organs and tissue were removed processed and wax embedded. A 12 week old R6/2 mouse plus wild type littermate was processed alongside for direct comparison. The 5 µm tissue sections were stained with haematoxylin and eosin (H&E) for tissue identification and immunostained with the S830 antibody for the identification of inclusions. Tissues and cell types in which inclusions were detected are summarised in Table 1 and examples are illustrated in Figure 1, Figure 2, Figure 3, Figure 4, and Figure 5. Inclusions were only detected in cell nuclei and in the majority of nuclei there was only one inclusion, but in some cell types: skeletal muscle fibres, liver hepatocytes and connective tissue fibroblasts, there were multiple nuclear inclusions, as in the R6/2 model. We noted considerable variation in the number of nuclear inclusions in the tissues examined between individual *Hdh* ^Q150/Q150^ mice but not in the overall distribution of inclusions.

**Table 1:** Distribution of polyQ inclusions in the peripheral tissues of HD mouse models.

Tissue/Organ	Inclusion – positive cells	*Hdh* ^Q150/Q150^	R6/2
Heart	Cardiac muscle fibres	+	+
Liver	Hepatocytes	++	++
	Bile duct epithelium	+	
Kidney	Tubule/duct epithelial cells	++	++
	Interstitial cells	++	++
	Glomerular cells	+	+
Adrenal gland	Zona glomerulosa	+	++
	Zona fasciculate	+	++
	Medulla	+++	+++
Stomach wall	Mucosal glands	++	++
	Submucosal plexus	++	++
	Myenteric plexus	++	++
	Serosal ganglia	++	++
	Smooth muscle fibres	+	+
Duodenum	Submucosal plexus	++	++
	Myenteric plexus	++	++
Rectum	Submucosal plexus	++	++
	Myenteric plexus	++	++
	Smooth muscle fibres	+	+
Pancreas	Endocrine - islets of Langerhans	++	+++
	Exocrine - acinar tissue	+	+
Skeletal muscle quadriceps	Muscle fibres	++	++
Skin	Nerves Schwann cell	+	+
Testicle	Seminiferous tubule epithelium	+	n/a
Seminal vesicle	Glandular Epithelium	++	++
Coagulation gland	Glandular Epithelium	++	++
Brown fat	Adipocytes	+	++
Connective tissue	Fibroblasts	+	+

Tissue/organ distribution of polyQ inclusions in the R6/2 and *Hdh*Q150 knock-in mouse models of HD. Frequency of inclusion is indicated as follows: + sparse; ++ moderate; +++ abundant.

### Distribution of nuclear inclusions in muscle, liver, and kidney

Nuclear inclusions were found in the skeletal muscle fibres (Figure 1A and 1B) of the *Hdh* ^Q150/Q150^ mice, with some nuclei containing up to 3 inclusions. Inclusions were also observed in the fibroblasts of the muscle connective tissue. In contrast nuclear inclusions were mostly absent from nuclei of the cardiac muscle fibres and were only observed in a few discrete patches of muscle fibres in the heart wall, a pattern that was identical to that observed in the R6/2 mouse. Nuclear inclusions were also apparent in fibroblasts in the connective tissue of the adventitia of larger cardiac blood vessels. Small numbers of inclusions were noted in the smooth muscle cells (see Figure 4E) of the stomach wall in a very patchy distribution in both mouse models.

In the liver, nuclear inclusions were found in the hepatocytes and connective tissue and many of the hepatocyte nuclei had multiple inclusions in a variety of sizes from 0.2 – 2 µm (Figure 1C and 1D). The distribution of inclusions in the hepatocytes was not uniform throughout the liver and staining was patchy. A very small number of Kupffer cells appeared to have inclusions in the nuclei, but identification of cell types in unstained tissue is difficult and a more detailed study will be needed to confirm the identity of these cells. One intrahepatic bile duct present on a slide of a liver preparation from the *Hdh* ^Q150/Q150^ mice contained inclusions in the epithelial cells (Figure 1E).

Inclusions were found throughout the kidney, in the cortex, medulla and papilla, in capsule fibroblasts, other connective tissue, interstitial cells, the epithelium of the tubules and ducts and in a few cells of the glomerulus (data not shown). It was not possible to identify which cell type had inclusions in the glomerulus, they may have been podocytes or mesangial cells. A more detailed study would be required to identify the cell type. Overall in the kidney, inclusions were found in low numbers but the numbers increased from the cortex to the papillae. The pattern was similar in both mouse models.

### Nuclear inclusions are most prominent in the medulla of the adrenal glands

The adrenal gland pathology has been well characterised in the R6/2 mice [15], [24]. In the adrenal glands of *Hdh* ^Q150/Q150^ mice (Figure 2A–2G) occasional nuclear inclusions were found in the fibroblasts of the gland capsule and very sparse nuclear inclusions in the zona glomerulosa and the zona fasciculata. In contrast there are many large inclusions in the nuclei of the chromaffin cells of the medulla. In R6/2 the distribution and numbers of nuclear inclusions in the connective tissue of the capsule and chromaffin cells of the medulla are similar but there are noticeably more nuclear inclusions in the cells of the zona glomerulosa and the zona fasciculata.

### Pancreatic nuclear inclusion pathology is conserved between the two mouse models, but is not accompanied by β-cell degranulation in the knock-in mice

In the endocrine pancreas, the principle cell type of the islets of Langerhans, the β-cells, contained nuclear inclusions (Figure 3A), but the percentage of cells with inclusions varied considerably between individual *Hdh* ^Q150/Q150^ mice and the overall size and appearance of the islets were similar to that of the controls (Figure 3B). Inclusions have been reported in the α-cells and δ-cells of the R6/2 islets [25] but without specialised staining procedures it was not possible to determine if the other islet cell types also contained inclusions in the knock-in model. A very small number of acinar cells of the exocrine pancreas were found to have nuclear inclusions and we noted inclusions in some cells of the connective tissue. The distribution of inclusions was similar in both animal models but the islet cells of R6/2 were smaller and more heavily stained. We also observed inclusions in epithelial cells of a pancreatic duct in R6/2, but did not find any ducts in the *Hdh* ^Q150/Q150^ sections examined for comparison. We examined the ultrastructure appearance of the pancreatic islets of the *Hdh* ^Q150/Q150^ mice (Figure 3C) and found no obvious differences at the ultrastructural level when compared to control animals (Figure 3D). This is in contrast to the previously described ultrastructure of the islets in R6/2 mice [25], in which there was a depletion in the numbers of β-granules in the β-cells and a reduction in the numbers of β-cells with a resultant reduction in the size of the islets, a result that we confirmed in this study (Figure 3E). The β-cells were small with fewer granules and organelles and the cytoplasm was denser than normal, suggestive of cell atrophy. The α-cells and δ-cells appeared relatively normal.

### Inclusion pathology is present throughout the stomach wall

We used transverse sections of the stomach wall of the *Hdh* ^Q150/Q150^ mice to investigate inclusion distribution in the fundus, corpus and pyloric regions of the stomach (Figure 4). Inclusion distribution was similar in all three regions. As in other tissues the distribution of inclusions was not uniform but patchy. In the gastric glands, small inclusions were found in the connective tissue and in the mucosal cells of the gland neck (Figure 4B). Larger inclusions were found in the epithelial cells at the gland base (Figure 4C), but specialised staining would be needed to establish the specific cell type involved: parietal, APUD or chief cells. They were also observed in connective tissue cells in the interstitium and adventitia of large blood vessels of the submucosa. There were large nuclear inclusions in the ganglionic neurons of the submucosal plexus (Meissner's) (Figure 4D) and in the ganglion neurons of the myenteric (Auerbach's) plexus (Figure 4F). We also noted nuclear inclusions in the neurons of a third group of ganglia, on the outer surface of the muscularis externa located mostly over the corpus region of the stomach, the serosal ganglia (Figure 4G) [26]. Nuclear inclusions were also observed in small groups of smooth muscle cells throughout the muscularis externa (Figure 4E). A similar distribution of inclusions was found in the R6/2 mouse model.

In the duodenum inclusions were observed in the ganglion cells of the myenteric and submucosal plexi and the interstitial connective tissue in both mouse models (data not shown). A similar distribution was seen in the rectum and we also observed a small number of smooth muscle cells with inclusions in the muscularis externa (data not shown). The ileum, jejunum, caecum and colon were not available for analysis.

### Inclusion pathology is comparable in the male and female reproductive organs of both the *Hdh*Q150 and R6/2 mouse models

The male reproductive organs of *Hdh* ^Q150/Q150^ mice contained a few cells in the testicular epithelium of the seminiferous tubules of the testis that had nuclear inclusions, the identity of which needs to be confirmed by appropriate histological staining. Inclusions were also noted in the peritubular contractile cells of the tunica propria. An examination of the seminal vesicles (Figure 5A) from both mouse models revealed the presence of inclusions in the glandular epithelial cells. Inclusions were also found in the glandular epithelial cells of the coagulation gland (dorsocranial part of the prostate gland) (Figure 5B) in both models. In females, inclusions were only found in the connective tissue of the oviduct and the ovarial capsule (tunica albuginea) and interstitium in both mouse models (data not shown)

### Selective inclusion pathology in adipose tissue

To investigate brown fat we examined the sub-clavical fat pads. A small number of the adipocyte nuclei were found to contain inclusions in *Hdh* ^Q150/Q150^ mice, larger numbers of inclusions were found in the R6/2 model. We examined the visceral fat pads and found no inclusions in white fat adipocytes in either mouse model (data not shown).

### Inclusion negative tissues

In addition to the ovaries and white fat already mentioned, inclusions were not present in any of the layers of the skin or principle cell types of the spleen in either mouse model. However, we observed small numbers of nuclear inclusions in connective tissue cells in the *Hdh* ^Q150/Q150^ and R6/2 mice in all tissues examined, including the fibroblasts of the adventitia of larger blood vessels. Even the tissues and organs in which the principle cell types did not contain inclusions, the connective tissue cells did, for example the myofibroblasts of the capsule and trabeculae in spleen (Figure 5C) and fibroblasts in subcutaneous connective tissue of the skin.

### Shrinkage/atrophy of organs in the *Hdh*Q150 knock-in mouse model

Symptomatic HD patients exhibit pronounced weight loss and muscle atrophy [17], [27], phenotypes that are modelled in both the R6/2 and *Hdh*Q150 mouse models of HD [10], [13], [28], [29]. Failure to gain weight begins at approximately 7 weeks of age in R6/2 males and 11 weeks in R6/2 females [28], [29] whereas, both male and female *Hdh* ^Q150/Q150^ mice stop gaining weight at approximately 9 months of age [13]. We have previously determined the extent of organ atrophy [15] in 12 week old symptomatic R6/2 mice for the heart, liver, kidneys, testes and skeletal muscle. In order to determine whether organ atrophy had also occurred in the knock-in model, we compared the weights of heart, kidney, quadriceps, spleen, liver and testes from four 22 month old *Hdh* ^Q150/Q150^ mice to those of four wild type littermates (two male and two female in each case) (Table 2). In all cases, the mean organ weight was lower in the *Hdh* ^Q150/Q150^ mice than in their wild type littermates. The difference in these values was highly statistically significant for quadriceps, kidney and testes but did not reach statistical significance for heart, liver and spleen. At least for liver and heart, this is most likely due to the small number of animals studied.

**Table 2:** Extent of organ atrophy in the *Hdh* ^Q150/Q150^ mice at 22 months of age.

	Mean Weight g (±Std. Error)		Independent samples *t*-test	% Weight of WT	% Weight of WT
Organ/Tissue	WT 22 month	*Hdh* ^Q150/Q150^ 22 month	2-tailed p values	*Hdh* ^Q150/Q150^ 22 month	R6/2 12 wk previous study [15]
Quadriceps muscle	0.17±0.01	0.09±0.01	p<0.001 *	53	42
Heart	0.18±0.03	0.12±0.01	p = 0.152	67	55
Liver	1.41±0.12	1.19±0.06	p = 0.212	84	75
Kidney	0.22±0.02	0.17±0.02	p = 0.031 *	78	50
Spleen	0.10±0.00	0.09±0.03	p = 0.391	90	67
Testes	0.08±0.01	0.03±0.01	p = 0.005 *	38	15

Organs were removed from perfusion fixed 22 month old *Hdh* ^Q150/Q150^ mice and their wild type littermate controls (n = 4 mice/genotype) and weighed to determine the extent of organ atrophy. These data are compared to those previously calculated for R6/2 mice at 12 weeks of age as compared to wild type littermates.

* statistically significant.

### Investigation of muscle fibre morphology

We had previously conducted a relatively detailed analysis of muscle pathology in the R6/2 mouse and had identified a uniform reduction in the muscle fibre diameter of the quadriceps muscles [15]. To investigate whether this is also a feature of *Hdh* ^Q150/Q150^ muscles, we measure the bound width (shortest) of all fibres in 10 random micrographs from transverse quadriceps sections stained with H&E from each mouse. The fibre diameters for each genotype were pooled *Hdh* ^Q150/Q150^, n = 881; wild type, n = 450. The mean diameters (±SE) were wild type, 44.36±14.46 and *Hdh* ^Q150/Q150^, 35.16±12.2 (p<0.001). The same micrographs were also examined for the percentage of fibres with central nuclei, the number of small angular fibres (Table 3) and the presence of split fibres. There were 10% central nuclei in the 22 month old *Hdh* ^Q150/Q150^ mice, which is greater than the normal value (≤3%) and a possible indicator of muscle myopathy [30] and split fibres were also present. The percentage of angular fibres was within the normal range and no other evidence for neuropathy was found.

**Table 3:** Analysis of indicators of myopathy and neuropathy in skeletal muscle.

	No. Fibres	No. Fibres Central Nuclei	% Fibres Central Nuclei	Total Angular Fibres	% Angular Fibres
WT	459	10	2.2	1	0.22
*Hdh* ^Q150/Q150^	916	99	10.81	5	0.54

Greater than 3% of central nuclei is considered abnormal and a possible indicator of muscle myopathy in humans. Based on these criteria, there is evidence for myopathy in the *Hdh* ^Q150/Q150^ mice. The percentage of angular fibres is within the normal range.

## Discussion

Although HD is a neurodegenerative disorder, the *HD* gene is ubiquitously expressed, and whilst it is understandable that HD research is predominantly focussed on the central nervous system (CNS), evidence is accumulating to suggest that some HD symptoms may be caused by a peripheral pathology. The mechanistic role that polyQ inclusions play in the pathogenesis of HD remains the subject of much debate, however, their presence is considered to be indicative of pathology. In this study, we have shown that the distribution of nuclear inclusions in the peripheral tissues of the R6/2 and *Hdh*Q150 models is almost identical at end-stage disease and therefore, the peripheral pathology of these two HD mouse models is highly comparable. Peripheral pathology was identified in skeletal muscle, heart, pancreas, adrenal gland, liver, kidney, the gastrointestinal (GI) tract, brown fat, male reproductive organs and connective tissue. In most cases, the cell types that are affected are, like neurons, terminally differentiated. Additional factors that might influence the propensity to form inclusions in a particular cell type could include expression level of the R6/2 transcript or *Hdh* gene, Htt proteolysis and protein folding and clearance networks. Organ atrophy was also a feature of both models with the testes exhibiting the most dramatic weight loss followed by skeletal muscle in both cases.

Therefore, we have demonstrated that the peripheral pathology documented in the R6/2 mice is not the result of the aberrant expression of a small N-terminal fragment of mutant Htt as it is also present in a genetically precise HD mouse model in which an expanded CAG repeat has been inserted into the mouse *Hdh* gene. This peripheral pathology may be responsible for some of the phenotypes exhibited by the two mouse models. The nuclear inclusions that form in brain have recruited many additional nuclear and cytoplasmic proteins and a detailed analysis of the proteins that are recruited into inclusions in any one of the peripheral tissues may shed light on the pathogenic processes. Whether peripheral pathology has relevance to the human disease is under investigation but, in general, has yet to be established.

Skeletal muscle atrophy is observed in many HD patients despite an adequate diet and food intake [27], significant body weight changes are measurable in early as well as late stage disease [17] and weight loss has recently been shown to track with CAG repeat length [18]. The use of a hand-held dynamometer has recently shown that people with HD have a significant reduction in lower limb muscle strength to, on average, about half of the strength of healthy matched controls [20]. The presence of dense granular deposits, immunoreactive to an anti-huntingtin antibody, have been found in the muscle of an HD patient, but were absent from a control sample [31]. Might the pathogenic mechanism that underlies these symptoms be intrinsic to muscle pathology? We have previously shown that nuclear inclusions form in the muscle fibres of R6/2 mice [15] and that the loss of muscle bulk in both the R6/2 and R6/1 mice is associated with pronounced muscle fibre atrophy. Perturbations in the R6/2 hypothalamic-pituitary-adrenal axis suggest that the R6/2 mice develop a Cushing-like syndrome [24] due to increased glucocorticoid levels, however, we did not observe the selective type 2 (fast) fibre type atrophy that this would predict, instead a pronounced uniform atrophy had occurred. A detailed study by Ribchester et al. also found no evidence for a myopathy (myofibre necrosis, myophagocytosis, regeneration, rounded atrophic fibres, fibre hypertrophy and splitting, centralized nuclei, fibrosis ring fibres, vacuoles or any sign of inflammation) [32] or for a denervation-like pathology (angulated atrophic fibres, fibre-type grouping, group atrophy or target fibres), discounting neuropathic causes until end-stage disease. Instead, they identified a complex phenotype with R6/2 muscle fibres undergo profound atrophy with many showing membrane characteristics of denervated or paralysed muscle fibres [32]. We have shown that the muscle fibres of the *Hdh*Q150 knock-in model, like those of the R6/2 mice contain nuclear inclusions. The evidence for myopathy in *Hdh* ^Q150/Q150^ mice was greater than in R6/2 as there was a higher frequency of central nuclei and some split fibres. In support of a mechanism intrinsic to muscle, a semi professional marathon runner at risk for HD developed signs of a slowly progressive myopathy with exercise-induced muscle fatigue, pain, elevated creatine kinase level and worsening of his running performance many years before the first signs of chorea were detected [33]. Common gene expression profiles in skeletal muscle between R6/2 mice, *Hdh* ^Q150/Q150^ mice and HD patients have previously been reported to reflect disease progression and be consistent with the beginnings of a transition from fast-twitch to slow twitch muscle fibre types [34]. More recently, impaired activity of PPARγ coactivator 1α (PGC-1α) and its target genes, important for inducing mitochondrial biogenesis and in influencing whether muscle contains slow twitch oxidative or fast twitch glycolytic fibres, has been demonstrated in the muscle and myoblasts of HD patients and the muscle of N171-82Q mice [35]. However, there is also evidence to suggest that the reduction in muscle bulk can occur by through pathogenic events exogenous to muscle that are either peripherally or centrally generated. The metabolic profile of plasma from both N171-82Q mice and HD patients supports a reduction in muscle bulk being caused by a catabolic state secondary to hypothalamic pathology [24], [36], [37] which is known to occur in both mouse models of HD and HD patients [24], [38], [39]. In addition, increased plasma cytokine levels, notably IL6 and TNFα, have been detected in people who carry the HD mutation 16 years before the predicted onset of motor symptoms [40] and could contribute to muscle wasting.

Early-onset cardiovasclular disease is the second leading cause of death in HD patients [41], [42]. The inclusion pathology in the hearts of both the R6/2 and *Hdh*Q150 models was comparatively sparse although cardiac dysfunction has been described in R6/2 mice [43]. The formation of polyQ amyloid has been shown to cause heart failure as transgenic mice that express an 83 residue long polyQ repeat in cardiomyocytes exhibited reduced cardiac function and dilation by five months and all died by eight months [44]. An electrolyte imbalance has been described in the R6/2 mice at 12 weeks of age [24] which could exacerbate cardiac failure. A detailed analysis of cardiac function in the *Hdh*Q150 knock-in model and in HD patients is warranted.

Pancreatic inclusion pathology has been reported in all of the N-terminal Htt transgenic HD models: R6/2 mice [15], [25], [45], [46], R6/1 mice (which express the same exon 1 transgene but at a lower level) [47] and N171-82Q mice [16] and the presence of aggregates has been shown to correlate with islet size [16]. A subset of R6/2 mice develop diabetes, and all R6/2 mice have impaired glucose homeostasis [45], [46], [48], [49], [50], a phenotype that has also been reported in the R6/1 [47], N171-82Q [16], [51] and *Hdh*Q150 knock-in models [34]. The pancreatic islets of the R6/2 mice have been shown to express reduced levels of the islet hormones: insulin, somatostatin and glucagon [25], [46], [48] and exhibit intrinsic deficits in insulin production [25], [46]. R6/2 mice develop an age-dependent reduction in insulin mRNA production and diminished expression of key regulators of insulin gene transcription: PDX-1, E2A, CBP, and p300 [46]. This is paralleled by a decrease in β-cell mass (possibly caused through a decrease in β-cell replication), an absence of exocytosis as induced by membrane depolarization and a dramatic reduction in the number of secretory vesicles in R6/2 β-cells [25]. A decrease in β-cell mass has also been described in R6/1 [47] and N171-82Q mice [16]. Further support for the diabetes in the R6/2 mice being caused by impairment in insulin release rather than insulin insensitivity has been generated by the administration of pharmacological agents [45]. In this study, we have shown that inclusions are present in the pancreatic islet cells of *Hdh* ^Q150/Q150^ mice which combined with previous reports of impaired glucose homeostasis in this model, suggests that the pancreatic pathology described in the N-terminal fragment models extends to the HD knock-in models. However, the inclusion pathology exhibited greater variation between *Hdh* ^Q150/Q150^ mice than in R6/2 and we did not find a reduction in the number of secretory vesicles in knock-in β-cells. The extent to which these findings extend to HD patients currently remains unclear. Impairment in insulin secretion capacity and a simultaneous decrease in insulin sensitivity, with an increase in the insulin resistance level was recently reported in normoglycaemic HD patients [21]. However, in a different study, the level of insulin transcripts as measured by immunostaining and the islet β-cell area were similar in HD patients and controls. Pancreatic islets appeared histologically normal and there was no sign of amyloid deposition [52].

Many of the peripheral symptoms that have been described in HD patients and mouse models of HD could be the result of disturbed hypothalamic-endocrine axes [53]. As mentioned above, disturbances in the hypothalamic-pituitary-adrenal axis could contribute to weight loss and there is marked adrenal pathology in both the R6/2 and *Hdh* ^Q150/Q150^ mice. The hypertrophy of the adrenal cortex and progressive increase in corticosterone levels could account for reduced bone mineral density, insulin resistance and muscular atrophy recorded in R6/2 mice [24]. Increased levels of corticosteroids have been reported in HD patients [24], [53], [54], but a recent study dissociated this from weight loss [54]. Impairments were[54] identified in several anterior pituitary axes [54]. Both central growth hormone (GH) and peripheral insulin growth factor-1 (IGF-1) somatotropic hormones were higher in HD patients, increased with disease progression and were linked to weight loss, potentially implicating a liver pathology. Testicular pathology has been described in HD patients with reduced numbers of germ cells and abnormal seminiferous tubule morphology [55]. We have shown that the testes are reduced in size in both R6/2 and *Hdh* ^Q150/Q150^ mice, also reported in the YAC128 HD mouse model [55], and testosterone levels are reduced in both R6/2 mice [56] and male HD patients [54], [57]. This could arise through a decrease in the production of gonadotrophin-releasing hormone in the hypothalamus [56], a loss of the direct neuronal hypothalamic-testicular pathway [54] or the direct effect of mutant Htt in the testes [55].

Body weight regulation is a highly complex process that involves the GI tract and adipose tissue in addition to the hypothalamus and pancreas discussed above [37]. It is governed by the balance of food intake, energy expenditure and metabolism. Both the R6/2 mice and HD patients have been shown to have an increased metabolic rate [36], [37]. The hypothalamus integrates peripheral signals of energy levels and regulates food intake, energy balance and metabolism. Van der Burg et al. [37] have shown that there are reduced levels of hypothalamic peptides important for regulating food intake and metabolism. We have demonstrated that inclusions are present in the adipocytes of R6/2 and *Hdh* ^Q150/Q150^ mice, a pathology also present in N171-82Q mice [58]. Adipose tissue-derived hormones that regulate food intake and glucose metabolism have been shown to be dysregulated in the R6/2 [50], [59] and *Hdh*Q140 knock-in HD models [59], suggesting an intrinsic adipocyte dysfunction that may contribute to weight loss. Mutant Htt has been shown to dysregulate gene expression in adipocytes, including PGC-1α and its target genes including the uncoupling proteins (UCPs) [37], [58], [59] resulting in impaired thermogenesis and metabolic abnormalities. As mentioned above, PGC-1α activity is also decreased in muscle from HD patients and N171-82Q mice [35] and given the pathology that we have described in R6/2 and *Hdh* ^Q150/Q150^ liver, an investigation of the UCPs in liver is warranted. We identified inclusion pathology throughout the stomach wall and in the plexi of the stomach, duodenum and rectum. Grehlin, a peptide produced in the gastric mucosa that stimulates food intake has been shown to be down-regulated in the R6/2 mice [37] implicating a direct role for the GI tract in HD pathology.

As peripheral tissues are more accessible than those of the CNS, the presence of peripheral HD pathologies might lead to biomarkers that can be used to track disease progression in therapeutic intervention trials. Whether peripheral clinical biomarkers for adult onset HD can be identified will depend on the analysis of patient samples. It is unlikely that inclusion formation in the peripheral tissues of the *Hdh*Q150 knock-in mice will provide a useful read-out in preclinical studies as our preliminary analyses suggest that aggregate formation in the periphery occurs later than that in brain [60]. However, the peripheral pathologies in the R6/2 model allow proof of concept studies with potentially disease modifying therapeutic compounds to be performed without the initial requirement to cross the blood brain barrier.

We have identified extensive inclusion pathology with a very similar distribution in two quite different mouse models of HD. There is mounting evidence to indicate that peripheral pathologies contribute to phenotypes exhibited by these and other HD mouse models alongside effects from the CNS. The size of the CAG repeat expansion in both the R6/2 and *Hdh*Q150 models is of a size that would cause childhood disease in humans and would be expected to result in a more widespread pathology than CAG repeat expansions in the adult onset range. Therefore, although many peripheral symptoms of adult onset HD have been documented, the extent to which the peripheral pathology identified in the mouse models relates to adult onset disease is as yet unknown. There have been very few studies of the peripheral pathology in HD organs, largely because *post mortem* specimens have not been available for analysis. However, it is very important that a comprehensive analysis of HD peripheral pathology is undertaken, both to allow a better mechanistic understanding of the disease and to inform possible therapeutic interventions that might alleviate some HD symptoms by targeting dysfunction in peripheral organs.

## Materials and Methods

### Ethics statement

All animal work was approved by the King's College London Ethical Review Panel and performed under Home Office project and personal licenses.

### HD mouse models

Hemizygous R6/2 mice [10] were bred by backcrossing R6/2 males to (CBA x C57Bl/6) F1 females (B6CBAF1/OlaHsd, Harlan Olac, Bicester, UK). *Hdh* ^Q150/Q150^ homozygous knock-in mice [12], [13] on a (CBA x C57Bl/6) F1 background were generated by intercrossing *Hdh* ^Q150/Q7^ heterozygous CBA/Ca and C57BL/6J congenic lines (inbred lines from Harlan Olac, Bicester, UK). All animals were subject to a 12 h light/dark cycle, had unlimited access to water and breeding chow (Special Diet Services, Witham, UK). Housing conditions and environmental enrichment were as previously described [29]. R6/2 mice were always housed with wild type mice. The CAG repeat size in the R6/2 mouse was 213 and in the *Hdh* ^Q150/Q150^ mice was 155±3.4 (SD).

### Genotyping and CAG repeat sizing

R6/2 and *Hdh* ^Q150/Q150^ mice were identified by PCR of tail-tip DNA. For R6/2, a 10 µl reaction contained 100 ng DNA, 1x Thermo-Start master mix (Thermo Scientific), 1 µl DMSO, 10 ng/µl forward primer 33727 [5′-CGCAGGCTAGGGCTGTCAATCATGCT-3′], and 10 ng/µl reverse primer 32252 [5′-TCATCAGCTTTTCCAGGGTCGCCAT-3′]. Cycling conditions were: 15 min @ 94°C, 35×(30 s @ 94°C; 30 s @ 60°C, 60 s @72°C) and 10 min @ 72°C. The amplified R6/2 transgene product was 272 bp. For *Hdh* ^Q150/Q150^ mice, a 20 µl reaction contained 150 ng tail-tip DNA, 0.1 mM dNTPs, 2 M betaine (Sigma), 1x Detloff buffer [15 mM Tris–HCl (pH 8.8), 15 mM Tris–HCl (pH 9.0), 16 mM (NH_4_)_2_SO_4_, 2.5 mM MgCl_2_, 0.15 mg/ml bovine serum albumin (BSA), 0.007% β-mercaptoethanol], 10 ng/µl forward primer MHD16 [5′-CCCATTCATTGCCTTGCTGCTAGG-3′], 10 ng/µl reverse primer MHD18 [5′-GACTCACGGTCGGTGCAGCGGTTCC-3′], and 1 U Herculase Taq polymerase (Stratagene). Amplification conditions were: 5 min @ 95°C, 30×(30 s @ 94°C, 30 s @ 58°C, 3 min @ 72°C) and 5 min @ 72°C. The wild type allele amplified a 278 bp product whereas the *Hdh*Q150 knock-in allele amplified a 707 bp product. Amplification of the CAG repeat from R6/2 mouse DNA was performed with a FAM labelled forward primer (GAGTCCCTCAAGTCCTTCCAGCA) and reverse primer (GCCCAAACTCACGGTCGGT) in 10 µl reactions containing: 0.2 mM dNTPs; 10% DMSO; AM buffer (67 mM Tris-HCL pH 8.8; 16.6 mM (NH_4_)S0_4_; 2 mM MgCl_2_; 0.17 mg/ml BSA) and 0.5U AmpliTaq DNA polymerase (Applied Biosystems). Cycling conditions were: 90 s @ 94°C, 24×(30 s @ 94°C; 30 s @ 65°C; 90 s @ 72°C) and 10 min @ 72°C. For *Hdh* ^Q150/Q150^ mice, the amplification reaction was as for genotyping (above) with a FAM labelled reverse MHD18 primer. All instruments and materials were obtained from Applied Biosystems unless indicated. The FAM-tagged PCR product (1 µl) together with MegaBACE^TM^ ET900 (Amersham Bioscience) internal size standard (0.04 µl) were denatured at 94°C, 5 min in 9 µl of HiDi-formamide and analyzed using an ABI3730 sequencer. Data analysis was performed using plate manager application GeneMapper v5.2- 3730XL.

### Histology

Peripheral tissues were dissected from, four 22 month old *Hdh* ^Q150/Q150^ mice, four wild type littermate control mice, one 12 week old R6/2 and one wild type littermate control, that had been perfusion fixed with 4% paraformaldehyde. The organs and tissues were removed for histological examination and the heart, kidneys, liver, spleen, quadriceps and testis weighed. Tissues were processed and embedded in paraffin wax and 5 µm sections were cut on an RM 2155 microtome (Leica Microsystems). A few sections from each tissue were stained with Harris's haematoxylin and eosin (H&E) [61].

### Immunohistochemistry

The remaining sections were immunoperoxidase stained with huntingtin antibody S830 [62]. Sections were dewaxed in histoclear (Agar Scientific), rehydrated, incubated in 4% hydrogen peroxide for 10 mins, washed in dH_2_0, antigen retrieved by pressure cooking on high in 0.1 M Citric acid pH 6.0 for 7 min and washed in dH_2_O [61]. Endogenous biotin was blocked using a Biotin Blocking kit (Vector Laboratories) and incubated in primary antibody (S830: sheep polyclonal; 1∶2000) in blocking solution (0.1 M Tris-HCl pH 7.5, 0.9% NaCl (TBS) and 2% bovine serum albumin (BSA)) at 4°C overnight, washed twice in TBS for 15 min and incubated for 1 h at RT in biotinylated horse anti-goat secondary antibody (Vector Laboratories) in blocking solution (1∶500). After washing with TBS, sections were incubated in ABC-HRP complex using the Vectastain Elite ABC kit (Vector Laboratories) as recommended by the manufacturer and then incubated with a 0.05% solution 3,3-diaminobezidine (DAB) in 0.1 M Tris–HCl pH 7.5. H_2_O_2_ was added to 0.03%, the colour allowed to develop for 10 min and the slices washed extensively in dH_2_O. Sections were counterstained with Methyl Green prior to viewing on an Axioskop 2 microscope (Zeiss) and images captured using an Axiocam camera, and Axiovision software (Zeiss).

### Morphometry

The H&E stained sections of the quadriceps muscle were examined and 10 random high power micrographs were taken for each animal. The images were then processed using Axiovision v4.6 software and the muscle fiber diameters estimated using the bound width measurement (minimum diameter).

### Electron microscopy

Pancreatic samples from three additional 22 month old *Hdh* ^Q150/Q150^ mice, 3 littermate controls and two 12 week old R6/2 mice were fixed in 2.5% glutaraldehyde in 0.125 M phosphate buffer and processed for transmission electron microscopical examination. 60 nm sections were cut and stained with saturated aqueous uranyl acetate and Reynolds lead citrate [63] and imaged using a Jeol JEM 1230 transmission electron microscope.

### Statistical analysis

Sample means were compared using the independent samples Student's *t*-test with the statistical package SPSS v 16.
